# Interactions Between Emodin and Efflux Transporters on Rat Enterocyte by a Validated Ussing Chamber Technique

**DOI:** 10.3389/fphar.2018.00646

**Published:** 2018-06-22

**Authors:** Juan Huang, Lan Guo, Ruixiang Tan, Meijin Wei, Jing Zhang, Ya Zhao, Lu Gong, Zhihai Huang, Xiaohui Qiu

**Affiliations:** ^1^The Second Clinical College of Guangzhou University of Chinese Medicine, Guangdong Provincial Hospital of Chinese Medicine, Guangzhou, China; ^2^Guangdong Provincial Key Laboratory of Clinical Research on Traditional Chinese Medicine Syndrome, Guangzhou, China

**Keywords:** emodin, P-gp, MRP2, MRP3, Ussing chamber technique

## Abstract

Emodin, a major active anthraquinone, frequently interacts with other drugs. As changes of efflux transporters on intestine are one of the essential reasons why the drugs interact with each other, a validated Ussing chamber technique was established to detect the interactions between emodin and efflux transporters, including P-glycoprotein (P-gp), multidrug-resistant associated protein 2 (MRP2), and multidrug-resistant associated protein 3 (MRP3). Digoxin, pravastatin, and teniposide were selected as the test substrates of P-gp, MRP2, and MRP3. Verapamil, MK571, and benzbromarone were their special inhibitors. The results showed that verapamil, MK571, and benzbromarone could increase digoxin, pravastatin, and teniposide absorption, and decrease their *E*_r_ values, respectively. Verapamil (220 μM) could significantly increase emodin absorption at 9.25 μM. In the presence of MK571 (186 μM), the *P*_app_ values of emodin from M-S were significantly increased and the efflux ratio decreased. With the treatment of emodin (185, 370, and 740 μM), digoxin absorption was significantly decreased while teniposide increased. These results indicated that emodin might be the substrate of P-gp and MRP2. Besides, it might be a P-gp inducer and MRP3 inhibitor on enterocyte, which are reported for the first time. These results will be helpful to explain the drug–drug interaction mechanisms between emodin and other drugs and provide basic data for clinical combination therapy.

## Introduction

Emodin (1,3,8trihydroxy-6-methylanthraquinone), a major active anthraquinone, is naturally present in some herbs which have been wildly used in Oriental countries, such as *Rheum officinale* Baill., *Polygonum multijiorum* Thunb., *Polygonum cuspidatum* Sieb. Et Zucc., etc. (Dong et al., 2016). In the past decades, emodin has been shown a wide spectrum of biological and pharmacological effects, such as hepatoprotective antiviral, anti-diabetic, anti-bacterial, anti-allergic, anti-osteoporotic, immunosuppressive, and neuroprotective activities (Dong et al., 2016; Monisha et al., 2016). Recent studies have placed emodin back into the limelight, which exhibits a good prospect in anticancer treatment with its anticancer activities against several types of cancer cells, such as lung carcinoma, gastric carcinoma, pancreatic cancer, and breast cancer, with apoptosis, anti-angiogenesis, and anti-proliferation as possible mechanisms of action (Heo et al., 2010; Wei et al., 2013; Li et al., 2014; Cha et al., 2015). Mitoxantrone, a commonly used anticancer drug, is the prodrug of emodin (Riahi et al., 2008).

In recent years, drug–drug interactions between emodin and other drugs attracted more and more researchers’ attention. Di et al. (2015) showed that piperine significantly improved the *in vivo* bioavailability of emodin and inhibited glucuronidation metabolism of emodin. Yu et al. (2017) demonstrated that 2,3,5,4’-tetrahydroxystilbene-2-β-D-glucoside could enhance the emodin absorption in a Caco-2 cell culture model. Meanwhile, many studies have shown synergistic effects between emodin and other antineoplastic drugs (Ko et al., 2010; Wang et al., 2010; Tan et al., 2011; Guo et al., 2013). Actually, changes of transporters on intestine affect the absorption characteristics of many drugs, which is one of the essential reasons why the drugs interact with each other. Some researchers have proved that emodin was the substrate of P-glycoprotein (P-gp) and multidrug-resistant associated protein 2 (MRP2; Teng et al., 2007, 2012; Liu et al., 2012). However, there were no reports about the research of emodin direct effects on intestinal transporters.

The Ussing chamber technique has been presently provided a physiologically relevant system for studying transepithelial transport of ion, drugs, and nutrients across various epithelial tissues. In this system, the drugs can be exposed at either mucosal or serosal levels, and therefore the absorption direction (M-S) and secretion direction (S-M) characteristics both can be detected. Furthermore, the usefulness of Ussing chambers for intestinal transport studies has long been recognized, and many researchers regard it as gold standards (Erik et al., 2016). Up to now, studies of P-gp influences in drug intestinal absorption by using this technique have been reported (Ballent et al., 2014; Xiao et al., 2016). However, there were few reports about testing and verifying this technique whether or not suitable for P-gp related studies. Moreover, other efflux transporters studies rarely reported by this technique.

In this study, a validated Ussing chamber technique was established and interaction studies between emodin and three efflux transporters including P-gp, MRP2, and multidrug-resistant associated protein 3 (MRP3) were carried out. To verify this Ussing chamber technique whether or not suitable for transepithelial transport studies related to efflux transporters, P-gp, MRP2, and MRP3 were employed as the typical proteins. Specific substrates and inhibitors were selected to detect P-gp, MRP2, and MRP3 activities and functions under our experimental conditions. Subsequently, interaction studies between emodin and these three efflux transporters were investigated. This is the first time to study the direct influence of emodin on rat intestinal P-gp, MRP2, and MRP3 functions by using this technique. It will be helpful to explain the drug–drug interaction mechanisms between emodin and other drugs and provide basic data for clinical combination therapy.

## Materials and Methods

### Chemicals and Reagents

Standards of digoxin and pravastatin were purchased from Chendu Rui Fensi Biotechnology Co., Ltd. (Chengdu, China). Benzbromarone and teniposide were obtained from Guangzhou Feibo Biotechnology Co., Ltd. (Guangzhou, China). Verapamil, topotecan, etoposide, and MK571 were obtained from Dalian Mellon Biotechnology Co., Ltd. (Dalian, China). Ginsenoside Rg_1_ and emodin were purchased from the National Institutes for Food and Drug Control (Beijing, China). Krebs’ Ringer bicarbonate (KRB) buffer is composed by 114 mM NaCl, 10 mM Glucose, 1.25 mM CaCl_2_⋅2H_2_O, 1.1 mM MgCl_2_⋅6H_2_O, 5.03 mM KCl, 0.30 mM NaH_2_PO_4_⋅H_2_O,1.65 mM Na_2_HPO_4_, and 25 mM NaHCO_3_ of pH 6.5 (Huang et al., 2016). Solvents were of HPLC grade and other chemicals used were of analytical grade.

### Animals

The specific pathogen-free male Sprague–Dawley rats weighing 220 ± 20 g were obtained from Guangdong Medical Laboratory Animal Center (Guangzhou, China). Before starting the experiments, the rats were kept in an environmentally controlled breeding room (temperature: 22 ± 1°C, humidity: 50–70%) and fed standard laboratory food and water for 1 week. The animals were fasted overnight with free access to water before experiments. All animal studies were approved by the Institutional Animal Ethics Committee of Guangdong Provincial Hospital of Chinese Medicine.

### Tissue Preparation

The four intestinal segments including duodenum, jejunum, ileum, and colon from anesthetized rats were immediately removed by surgery and washed with KRB solution. Each section of the intestinal segments was then placed in cold (on ice), bubbled (O_2_:CO_2_ 95:5) KRB buffer. The four intestinal segments were then cut into 2 cm pieces, respectively, and serosas were removed by blunt dissection. Peyer’s patches were excluded from the experiment by visually identified. The stripped tissues were mounted in chambers with exposed surface area of 0.49 cm^2^. Temperature of the chambers was maintained at 37 ± 0.5°C. Each of the half-cells in the chambers was filled with 5 mL fresh KRB buffer (pH 6.5). Tissue viability was continuously controlled by potential difference (PD). Tissue with electrical values less than 2 mV was refused (Moazed and Hiebert, 2007; Heinen et al., 2013).

### Model Validation for Efflux Transporters’ Studies

Digoxin, pravastatin, and teniposide were employed as the test substrates of P-gp, MRP2, and MRP3. Verapamil, MK571, and benzbromarone were inhibitors of these three efflux transporters (**Table 1**; Kool et al., 1999; Zhou et al., 2008; Ellis et al., 2013; Abbasi et al., 2016; Liu et al., 2017). The experiments were started after 30 min equilibration time, by changing the KRB buffer on both sides; 5 mL KRB solution with different test compounds was filled in the mucosal or serosal compartment; meanwhile, equal volume fresh buffer was added to the other compartment. In the inhibitory studies, the inhibitors verapamil, MK571, and benzbromarone were added to the mucosal side, respectively; 0.5 mL samples were withdrawn from the receiver compartment every 30 min and replaced with fresh KRB buffer. The bidirectional transport studies with specific inhibitors were assessed to validate this model whether or not suitable for P-gp, MRP2, and MRP3 studies. Samples were stored at –80°C until analysis (Deusser et al., 2013).

**Table 1:** The substrates and inhibitors of P-gp, MRP2, and MRP3.

	SRM settings – ion pairs (*m/z*)				
	substrates		IS		Inhibitors
P-gp	Digoxin	779.147→475.447, 649.548	Ginsenoside Rg1	799.400→637.600	Verapamil
MRP2	Pravastatin	423.232→303.196, 321.138	Topotecan	422.082→320.016, 377.042	MK571
MRP3	Teniposide	674.241→383.235	Etoposide	606.213→229.242	Benzbromarone

*The SRM settings of the substrates and specific internal standards (ISs) were performed by LC-MS/MS system.*

### Interaction Studies Between Emodin and Efflux Transporters

In this study, the effects of the three efflux transporters on emodin intestinal absorption were investigated. Meanwhile, the influence of emodin on intestinal P-gp, MRP2, and MRP3 functions were also discussed. Solutions with three concentrations (low, middle, and high) of emodin were filled in the mucosal or serosal compartment to measure the bidirectional transport characteristic. In order to observe whether or which efflux transporters involved in emodin intestinal absorption process, verapamil, MK571, and benzbromarone were added to the mucosal side, respectively. In the emodin impact experiments, digoxin, pravastatin, and teniposide were employed as P-gp, MRP2, and MRP3 substrates. Changes of the bidirectional transport characteristic were measured before and after adding emodin to the mucosal side.

### Preparation of Perfusate Samples

Frozen samples were adequately vortexed after thawing at the room temperature; 10 μL suitable IS solution was added to 200 μL samples; 200 μL methanol was added after a thorough vortex mixing for 30 s. The mixtures were then vortexed for 30 s and centrifuged at 15,000 rpm for 30 min. Finally, 5 μL of supernatant was injected into the LC-MS/MS system.

### Liquid Chromatographic and Mass Spectrometric Conditions

The quantifications of the compounds were performed by the TSQ Quantum Ultra Triple Quadrupole LC-MS/MS system from Thermo Fisher Scientific. Chromatography was carried out using an Agilent Poroshell 120 SB-C18 (2.7 μm, 2.1 mm × 100 mm) column with a Phenomenex AFO-8497 C_18_ pre-column, operating at 30°C. Adaptive gradient elution methods were applied for the analytes. The flow rate of the mobile phase was kept at 0.2 mL min^-1^. Flow was directed to the ion spray interface. All measurements were carried out in negative ESI mode. Ion spray voltage was -2500 V. Vaporizer and capillary temperatures were set at 250 and 350°C, respectively. Auto sampler temperature was set at 10°C. Sheath gas and aux gas were set at 30 and 15 Arb, respectively. The selective reaction monitoring (SRM) transitions were at *m/z* 268.934→225.047 for emodin and *m/z* 779.147→475.447 for ginsenoside Rg_1_ [internal standard (IS)]. Other SRM settings and specific IS are shown in **Table 1**. Peak integrations and calibrations were carried out using LC Quan 2.5.2 software from Thermo Fisher Scientific. All the data were within the acceptable limits to meet the guidelines for bioanalytical methods.

### Data Analysis

The *Q* (accumulative quantity), *P*_app_ (apparent permeability), *E_r_* (efflux ratio), *P*_r_ (*P*_app_ ratio), and *E_rr_* (*E_r_* ratio) across the excited rat intestinal segments in the Ussing chamber were calculated using the following equations (Li et al., 2012; Huang et al., 2016):

(1) $${Q=5C}_{n}+0.5\sum_{i=1}^{n-1}C_{i}$$

(2) $$P_{\mathrm{app}}=\frac{dQ/dt}{A\cdot C_{0}}$$

(3) $$E_{r}=\frac{P_{S-M}}{P_{M-S}}$$

(4) $$P_{r}{=P}_{\mathrm{app}}{/P}_{\mathrm{app}\;(\mathrm{control})}$$

(5) $$E_{\mathrm{rr}}{=E}_{r}{/E}_{r\;(\mathrm{control})}$$

where *C*_n_ (nM) is the concentration of the drug at the time point (*n*), *Q* (nM) is the accumulated absorption amount, *A* is the exposed surface area of the intestine (0.49 cm^2^), *dQ*/*dt* (nM⋅s^-1^) is the amount of the drug transported, and *C*_0_ (nM) is the initial concentration of the test drug. Experiments were performed in batches, so control groups were set as one for each batch.

### Statistical Analyses

The data are presented as the mean ± SD for all experiments. Independent samples *t*-test was applied to compare the means between treatments. One-way ANOVA with LSD (equal variances assumed) or Danett T3 (equal variances not assumed) multiple comparison (*post hoc*) tests were used to evaluate statistical differences. A *p*-value of less than 0.05 was considered statistical significance.

## Results

### The PD Values for Rat Intestinal Segments

After 30 min equilibration time, PD was 7.16 ± 1.59, 7.23 ± 2.18, 6.50 ± 0.75, and 4.12 ± 0.69 mV for duodenum, jejunum, ileum, and colon, respectively. Obviously, the PD values were higher for small intestine segments under this experimental condition. In general, the PD slightly decreased by time when the chambers with small amount drugs (<200 μM).

### Model Validation for Efflux Transporters’ Studies Were Employed as the Test Substrates of P-gp, MRP2, and MRP3

**FIGURE 1:**
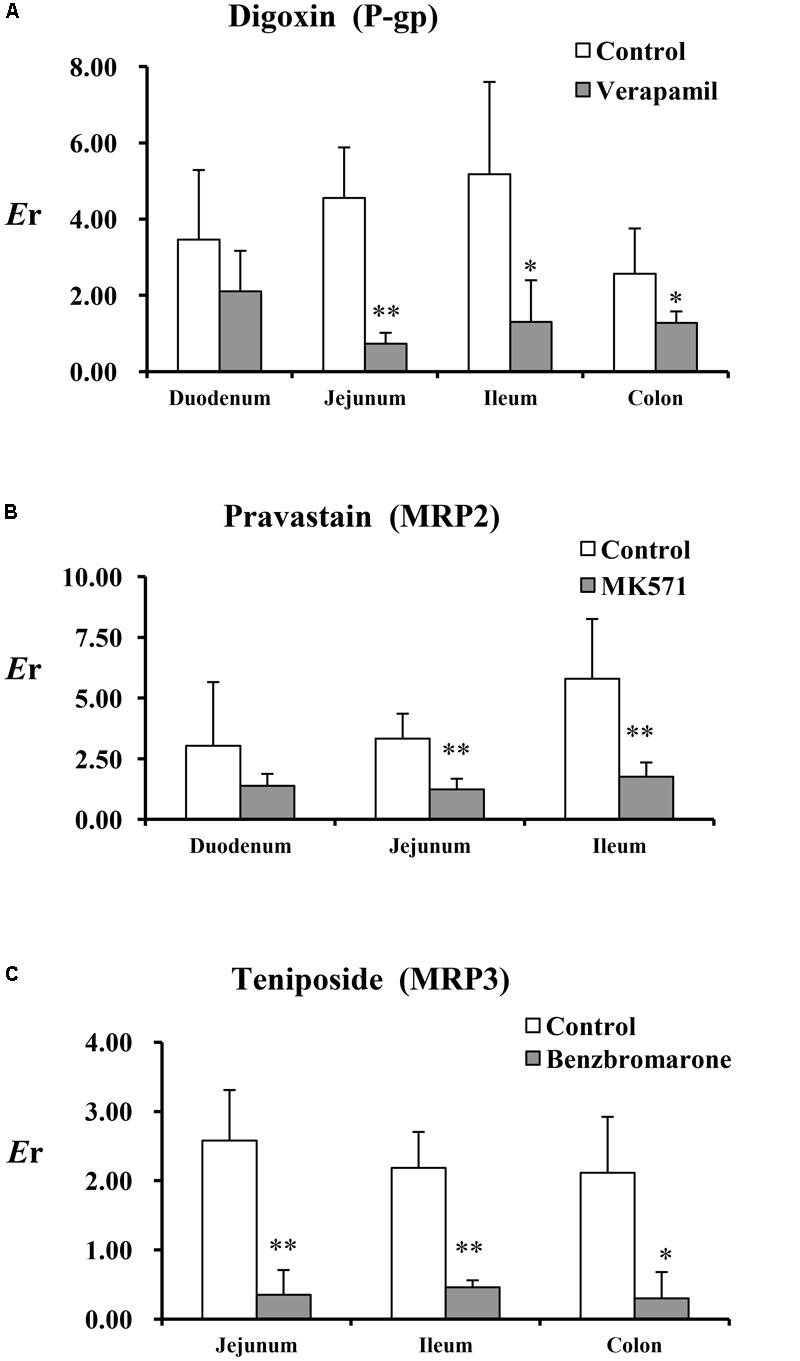
The *E*r of the test substances in perfused rat intestinal segments. **(A)** Digoxin, **(B)** pravastatin, and **(C)** teniposide were employed as the test substrates of P-gp, MRP2, and MRP3. Verapamil, MK571, and benzbromarone were inhibitors of P-gp, MRP2, and MRP3. Each bar represents the mean with SD of five measurements (^∗^*p* < 0.05 and ^∗∗^*p* < 0.01, compared to the control according to independent samples *t*-test).

The *P*_app_ and *E*r values of the test substances (digoxin, pravastatin, and teniposide) are summarized in **Table 2** and **Figure 1**. The results showed that the inhibitors, including verapamil, MK571, and benzbromarone, could significantly increase the absorption of the aforesaid substances and inhibit their secretion, and thus their *E*r values were decreased. In P-gp validation study, the concentrations of the inhibitor (verapamil) were set at 220 and 440 μM for the small intestine segments and colon, respectively. It was observed that verapamil could significantly inhibit the effect of P-gp protein on jejunum, ileum, and colon. However, it was showed no statistic difference on duodenum after adding the inhibitor. These may be related to the distribution characteristic of P-gp on rat enterocyte. In MRP2 and MRP3 validation studies, the inhibitors MK571 (186 μM) and benzbromarone (470 μM) were also displayed significant inhibition effect on both jejunum and ileum. These results indicated that the Ussing chamber technique could be used to investigate the role of P-gp, MRP2, and MRP3 in drug intestinal transport studies. Though the distribution characteristics of the effluxes were different, both jejunum and ileum were suitable for the study. Finally, jejunum was chosen for further studies because of the most stable data it revealed during model validation research.

**Table 2:** The *P*_app_ of the test substances in perfused rat intestinal segments.

*P*_app_ (× 10^-5^ cm/s)	Segments	M-S		S-M	
		Control	With inhibitor	Control	With inhibitor
Digoxin (P-gp)	Duodenum	7.25 ± 3.28	4.53 ± 2.32	18.55 ± 14.38	7.97 ± 3.22
	Jejunum	5.78 ± 1.12	11.86 ± 5.85*	26.96 ± 12.49	9.64 ± 8.64*
	Ileum	4.31 ± 1.50	19.12 ± 9.05*	22.78 ± 13.60	15.71 ± 10.82
	Colon	6.31 ± 3.31	18.94 ± 3.43**	13.70 ± 6.63	19.11 ± 4.01
Pravastatin (MRP2)	Duodenum	12.45 ± 6.73	7.79 ± 1.48	28.73 ± 16.52	11.25 ± 5.27
	Jejunum	14.62 ± 3.94	10.70 ± 2.68	49.60 ± 22.56	12.72 ± 3.34**
	Ileum	12.23 ± 5.25	12.45 ± 6.79	67.84 ± 32.52	22.45 ± 14.09**
Teniposide (MRP3)	Jejunum	1.40 ± 0.44	3.72 ± 0.97**	3.35 ± 1.40	1.31 ± 0.64**
	Ileum	1.26 ± 0.77	2.80 ± 1.95	2.45 ± 1.00	1.03 ± 0.29*
	Colon	1.60 ± 1.11	2.83 ± 0.84	2.13 ± 1.19	0.83 ± 0.19*

*Digoxin (25 μM), pravastatin (47 μM), and teniposide (60 μM) were employed as the test substrates of P-gp, MRP2, and MRP3. Verapamil (220 μM), MK571 (186 μM), and benzbromarone (470 μM) were inhibitors of P-gp, MRP2, and MRP3. Each column represents the mean with SD of five measurements (^∗^*p* < 0.05 and ^∗∗^*p* < 0.01, compared to the control according to independent samples *t*-test).*

### The Absorption Characteristics of Emodin

**FIGURE 2:**
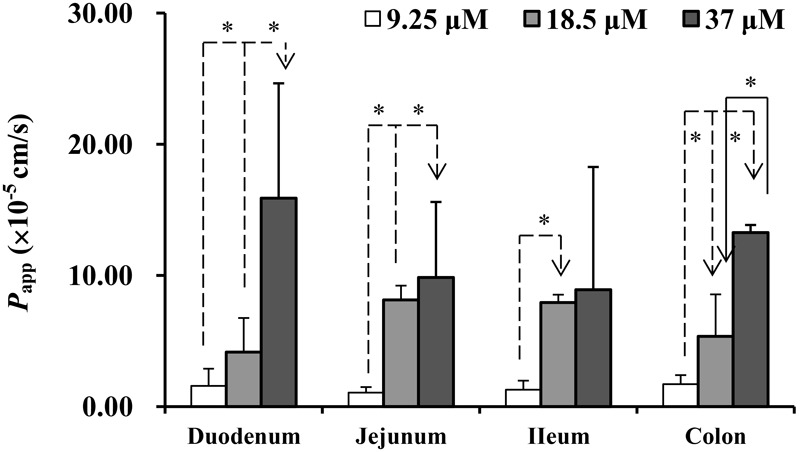
The *P*_app_ of emodin from mucosal to the serosal side in different intestinal segments. Experiments were conducted at three concentrations (9.25, 18.5, and 36.5 μM). Each bar represents the mean with SD of five measurements (^∗^*p* < 0.05, statistically significant differences among different concentrations, according to a one-way ANOVA test).

**FIGURE 3:**
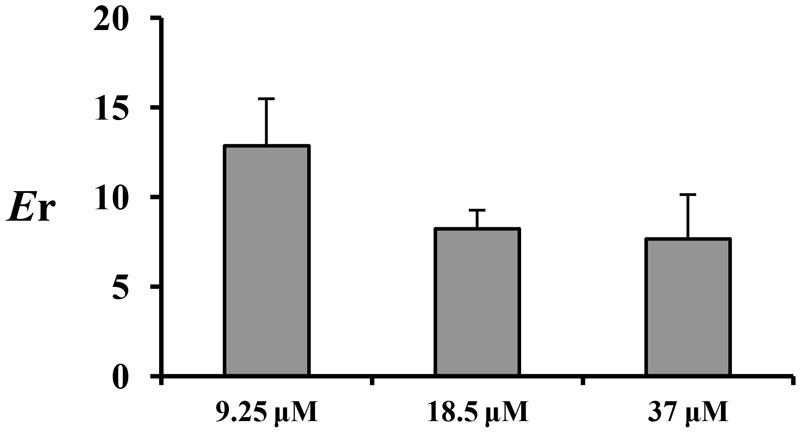
The *E*_r_ of emodin in jejunum at three concentrations (9.25, 18.5, and 37 μM). Each bar represents the mean with SD of five measurements.

The absorption characteristics of emodin at three concentrations (9.25, 18.5, and 37 μM) in different intestinal segments were investigated. The intestinal absorption rates of emodin displayed no regioselectivity whereas the *P*_app_ values from M-S were very low at the lowest concentration (**Figure 2**). These indicated that some efflux transporters may involved in emodin intestinal transport. The *E*r values of emodin in jejunum were all more than five at the three concentrations, pointing out that efflux transporters were involved in emodin intestinal absorption (**Figure 3**).

### Interaction Studies Between Emodin and Efflux Transporters

**FIGURE 4:**
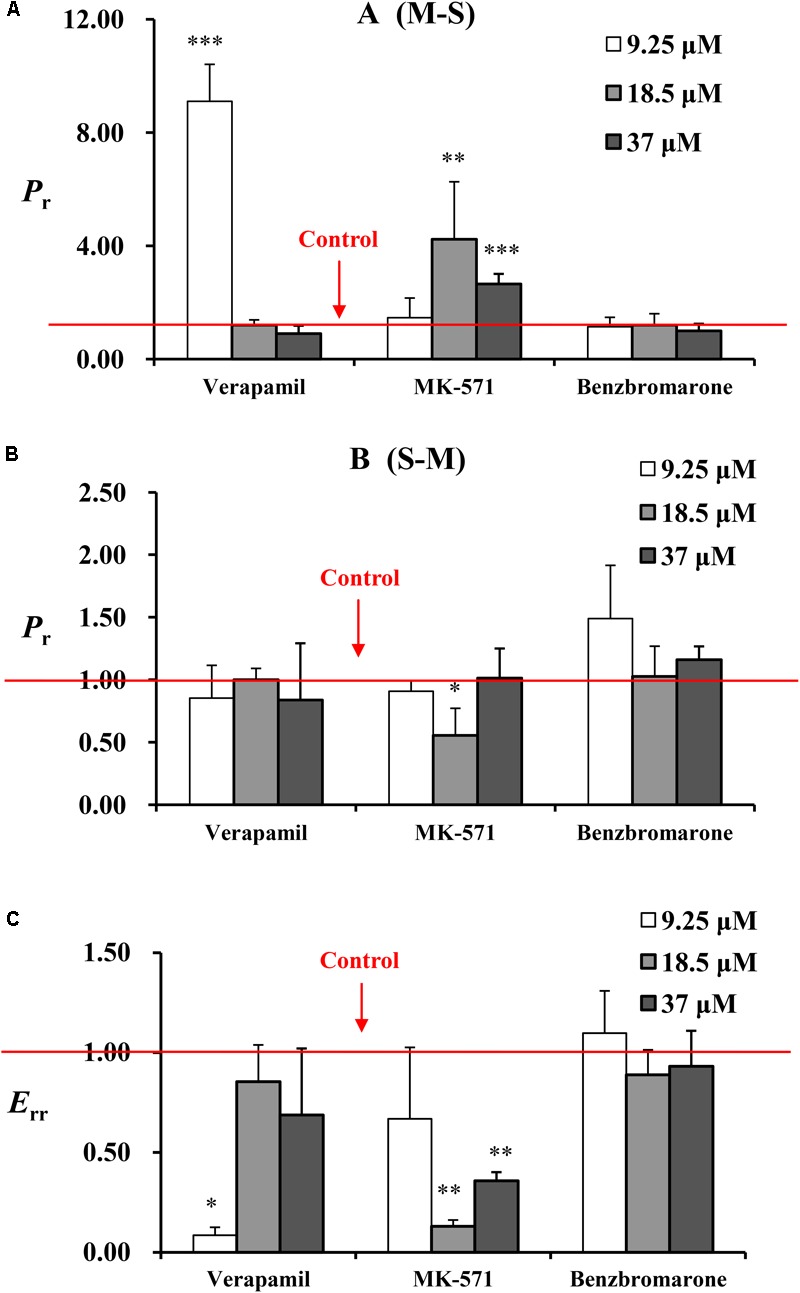
The *P*_r_ from **(A)** M-S and **(B)** S-M and **(C)** *E*_rr_ of emodin at three concentrations (9.25, 18.5, and 37 μM) in jejunum. Experiments were performed in the presence of verapamil (220 μM), MK571 (186 μM), and benzbromarone (470 μM). The three inhibitors were loaded onto the mucosal side, respectively. For the experiments were performed in batches, control groups were set as one for each batch. The result represents the ratio of *P*_app_ or *E*_r_ between without or with inhibitor. Each bar represents the mean with SD of five measurements (^∗^*p* < 0.05, ^∗∗^*p* < 0.01, and ^∗∗∗^*p* < 0.001, compared to the control according to independent samples *t*-test).

Transport studies were performed in the presence of P-gp, MRP2, and MRP3 inhibitors (verapamil, MK571, and benzbromarone) to determine the effect of these effluxes on the transport of emodin. The data are summarized in **Figure 4**. Verapamil (220 μM) could markedly increase emodin absorption and decrease the efflux ratio at 9.25 μM. In the presence of the MRP2 efflux transporter inhibitor MK571 (186 μM), the *P*_app_ values of emodin from mucosal to the serosal side were significantly increased and the efflux ratio decreased. However, no significant differences were observed in the presence of benzbromarone. These results indicated that emodin might be the substrate of P-gp and MRP2.

**FIGURE 5:**
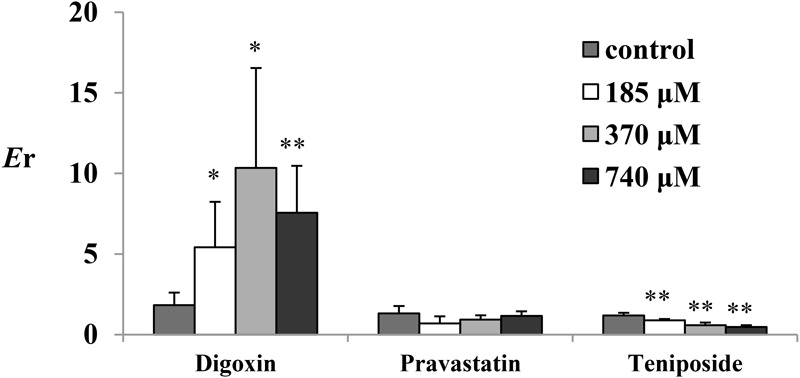
The *E*_r_ of digoxin (25 μM), pravastatin (47 μM), and teniposide (60 μM) in the presence of emodin. Emodin was loaded onto the mucosal side at three concentrations (185, 370, and 740 μM). Each bar represents the mean with SD of five measurements (^∗^*p* < 0.05 and ^∗∗^*p* < 0.01, compared to the control according to independent samples *t*-test).

To investigate the role of emodin on the efflux transporters P-gp, MRP2, and MRP3 functions on rat enterocyte, digoxin, pravastatin, and teniposide were carried out for transport studies in the presence of emodin. With the treatment of emodin, the *P*_app_ values of digoxin from M-S significantly decreased while those from S-M increased. Thus, the *E*_r_ values of digoxin were significant higher after adding emodin. The date are summarized in **Table 3** and **Figure 5**. These results indicate that emodin could enhance P-gp function on rat jejunum. In the presence of emodin, the *P*_app_ from S-M and *E*r of the MRP3 substrate teniposide remarkably decreased, indicating that emodin might be an MRP3 inhibitor. No significant differences were observed in the *P*_app_ and *E*_r_ values of pravastatin after emodin added.

**Table 3:** The *P*_app_ from M-S and S-M of digoxin (25 μM), pravastatin (47 μM), and teniposide (60 μM) in the presence of emodin.

*P*_app_ (×10^-5^ cm/s)		Control	Emodin concentration (μM)		
			185	370	740
Digoxin	M-S	9.16 ± 4.10	5.58 ± 2.98	4.90 ± 1.28	2.04 ± 0.76**
	S-M	15.19 ± 4.39	25.66 ± 4.76*	46.09 ± 19.06*	14.16 ± 2.97
Pravastatin	M-S	6.45 ± 2.79	12.28 ± 1.99	10.63 ± 4.03	9.38 ± 2.87
	S-M	8.22 ± 4.04	7.03 ± 1.98	9.08 ± 1.27	10.48 ± 2.73
Teniposide	M-S	6.94 ± 1.75	6.32 ± 0.59	5.25 ± 0.73	9.11 ± 2.73
	S-M	8.18 ± 1.59	5.61 ± 0.57*	3.12 ± 1.32**	4.11 ± 0.62**

*Emodin was loaded onto the mucosal side at three concentrations (185, 370, and 740 μM). Each column represents the mean with SD of five measurements (^∗^*p* < 0.05 and ^∗∗^*p* < 0.01, compared to the control according to a one-way ANOVA with *post hoc* test).*

## Discussion

Emodin, a potential antineoplastic drug, has been proved to have drug–drug interactions with many other drugs whereas the mechanisms have not yet to be discovered. Our results showed that emodin was the substrate of P-gp and MRP2, which is consistent with the literature reported (Teng et al., 2012; Zhang et al., 2012). Furthermore, we found that emodin might be a P-gp inducer and MRP3 inhibitor on enterocyte, which has not been reported in the literature before. However, verapamil only promoted emodin intestinal absorption at a low concentration; this is probably due to the fact that emodin itself is a P-gp inducer. MRP3, an efflux transporter, mainly distributes in liver, intestine, and adrenal glands, while most researches focused on its function and expression on liver. It is involved in the enterohepatic circulation of non-sulfated and sulfated bile salts such as glycocholates and taurocholates (Keppler, 2014, 2017). Our results showed that emodin might not be a substrate of MRP3 whereas it could inhibit MRP3 function on enterocyte, which indicated that the role of MRP3 should not be ignored in intestine. We consider that emodin might regulate MRP3 function by influencing the upstream proteins or kinases. Furthermore, we believe that oral administration of emodin would affect bile transport to some extent.

In this study, we used the Ussing chamber technique to evaluate the interactions between emodin and efflux transporters, which is regarded as gold standards for drug transport studies. The use of a living and intact intestinal tissue is more realistic than cell cultures and provides many advantages. The intestinal tissues are likely to express all the transporters and the enzymes at the same “physiological” level of expression. Moreover, the data obtained through rat intestine can be directly correlated to *in vivo* experiments that will be conducted in the same animal model (Mazzaferro et al., 2012; Sjögren et al., 2016). However, samples withdrawn from the receiver compartment often at very low concentration. With the development of LC-MS/MS technology, the amount of test compounds can easier to be detected. Besides, the procedure for tissue preparation is a technically challenging technique. KRB solution should be filtered with 0.22 μm filter and the removed intestine tissue should be placed in the cold KBR solution with gas in incessancy. Preparation must be done on ice carefully for it takes time and is associated with risks of tissue damage.

Although emodin has multiple pharmacological activities, its toxicity has attracted more and more attention in recent years. However, emodin treatment is a double-edged sword. It showed protective effect on alcoholic liver injury while hepatotoxicity appeared with high doses and long-term drug delivery (Dong et al., 2009; Wang et al., 2011; Liu et al., 2014). We suspect that the changeable role of emodin may be related to the variation of intestinal environment, including intestinal transporters, structures, microorganisms, and so on. Therefore, it requires further study to find out the changes of environment *in vivo* and emodin disposition characteristics during long-term administration process.

## Conclusion

In the present study, we have shown that the Ussing chamber technique was suitable for P-gp, MRP2, and MRP3 related studies on rat enterocyte. On the basis of this technique, we discovered that emodin might be the substrate of P-gp and MRP2, but not MRP3. Besides, emodin could decrease digoxin and increase teniposide absorption on rat intestine, indicating that emodin might be a P-gp inducer and MRP3 inhibitor.

## Author Contributions

JH and XQ designed the project. JH, LaG, RT, and MW performed the experiments. JH, LaG, RT, and XQ analyzed the data. JH, JZ, YZ, LuG, ZH, and XQ wrote the manuscript.

## Conflict of Interest Statement

The authors declare that the research was conducted in the absence of any commercial or financial relationships that could be construed as a potential conflict of interest.
